# A minimum price per unit of alcohol: A focus group study to investigate public opinion concerning UK government proposals to introduce new price controls to curb alcohol consumption

**DOI:** 10.1186/1471-2458-12-1023

**Published:** 2012-11-23

**Authors:** Adam J Lonsdale, Sarah J Hardcastle, Martin S Hagger

**Affiliations:** 1 Department of Psychology, Oxford Brookes University, Oxford, OX3 0BP, UK; 2 School of Sport and Service Management, University of Brighton, Eastbourne, BN20 7SR, UK; 3 School of Psychology and Speech Pathology, Curtin University, GPO Box U1987, Perth, WA6845, Australia

## Abstract

**Background:**

UK drinkers regularly consume alcohol in excess of guideline limits. One reason for this may be the high availability of low-cost alcoholic beverages. The introduction of a minimum price per unit of alcohol policy has been proposed as a means to reduce UK alcohol consumption. However, there is little in-depth research investigating public attitudes and beliefs regarding a minimum pricing policy. The aim of the present research was to investigate people’s attitudes and beliefs toward the introduction of a minimum price per unit of alcohol policy and their views on how the policy could be made acceptable to the general public.

**Methods:**

Twenty-eight focus groups were conducted to gain in-depth data on attitudes, knowledge, and beliefs regarding the introduction of a minimum price per unit of alcohol policy. Participants (total N = 218) were asked to give their opinions about the policy, its possible outcomes, and how its introduction might be made more acceptable. Transcribed focus-group discussions were analysed for emergent themes using inductive thematic content analysis.

**Results:**

Analysis indicated that participants’ objections to a minimum price had three main themes: (1) scepticism of minimum pricing as an effective means to reduce harmful alcohol consumption; (2) a dislike of the policy for a number of reasons (e.g., it was perceived to ‘punish’ the moderate drinker); and (3) concern that the policy might create or exacerbate existing social problems. There was a general perception that the policy was aimed at ‘problem’ and underage drinkers. Participants expressed some qualified support for the policy but stated that it would only work as part of a wider campaign including other educational elements.

**Conclusions:**

There was little evidence to suggest that people would support the introduction of a minimum price per unit of alcohol policy. Scepticism about the effectiveness of the policy is likely to represent the most significant barrier to public support. Findings also suggest that clearer educational messages are needed to dispel misconceptions regarding the effectiveness of the policy and the introduction of the policy as part of a package of government initiatives to address excess alcohol consumption might be the best way to advance support for the policy.

## Background

Excess alcohol consumption is known to have significant health, economic, and social consequences for people in the UK. Consumption of alcohol in excess is associated with increased risks of chronic health problems such as heart disease, liver cirrhosis, and some cancers [1-4]. The emergency treatment of alcohol-related injuries and hospital admissions are known to place a considerable burden on UK health care costs. A recent estimate suggested that the treatment of alcohol-related problems cost the UK National Health Service (NHS) £2.7 billion per annum [5]. There are also numerous maladaptive social consequences such that great numbers of people are frequently affected indirectly by the behaviour of others who drink alcohol to excess (e.g., domestic violence, street disorder, and criminal behaviour). The annual cost to the UK taxpayer of dealing with the secondary consequences of excessive alcohol consumption (so-called ‘passive’ drinking) [6] is estimated at £7.3 billion through the provision of policing, administration of the justice system, incarceration, and rehabilitation costs.

The problems associated with alcohol misuse are further exacerbated by evidence that alcohol consumption in the UK increased by 40% between 1970 and 2007, in contrast to falling consumption in many other European nations [7]. A recent survey found that 40% of men and 33% of women in the UK drink above guideline limits at least once a week. Evidence indicates that the rise in alcohol consumption can be attributed to several factors: (1) increases in alcohol consumption among certain groups (e.g., young people and women) who did not previously drink to excess [8]; (2) increased availability and affordability of alcohol [9]; and (3) the advent of aggressive marketing by the alcohol industry [10].

In recognition of these problems, the UK government has investigated possible legislative solutions to curb alcohol consumption and policies to raise the price of alcohol have been at the forefront of these solutions. Such approaches are based on clear evidence that raising the cost of alcohol leads to concomitant reductions in alcohol consumption. For example, a comprehensive meta-analysis of studies on pricing and alcohol consumption reported a significant effect of alcohol pricing on alcohol consumption [11]. The research also revealed a reduction in mortality from chronic illness associated with excessive alcohol consumption and in the negative social effects of alcohol including violent crime, social disorder, and accidents. The majority of studies have focused on the use of governmental taxation and duty to raise the price of alcoholic beverages. However, one of the disadvantages of taxation is that it applies uniformly to all alcoholic beverages which tends to maintain the disparity between the cost of alcohol at the high- and low-ends of the market [12]. Therefore, tax increases notwithstanding, there is still considerable scope for heavily-discounted alcoholic beverages to be available in retail outlets that have small margins and sell strong alcoholic beverages in bulk. In addition, taxation does not curb retailers from selling discounted alcohol via multi-buy promotions such as ‘buy-one-get-one-free’ or ‘happy hours’. This means that at the low-end of the alcoholic beverages retail sector is still able to produce relatively low-cost alcohol.

The introduction of a pricing policy that is based on the alcohol content (or ‘strength’) of beverages such as a minimum price per unit of alcohol policy has been put forward as a possible solution [13]. Systematic reviews of research concerned with the economics of drinking behaviour have demonstrated strong links between the price of alcohol and consumption levels [11,14-17]. One recent review concluded that “public policies that raise prices of alcohol are an effective means to reduce drinking” [11], p. 179]. The evidence base for a proposed minimum price per unit is informed by a series of reports endorsed by the UK Department of Health and Scottish Government providing economic models that predict significant reductions in alcohol consumption through the introduction of a minimum price per 10ml ‘unit’ of alcohol [10,18]. Based on the most recent estimates from Scottish modelling data, introducing a 40 pence-per-unit minimum would reduce alcohol consumption by 1.9% and a 50 pence-per-unit minimum by 5.7% [18]. The reviews also indicated that a 50p minimum price would be worth savings of £793.7 million in terms of the overall costs associated with treating and managing excess alcohol consumption [10]. In addition, a recent case study in Canada has provided the best evidence yet that minimum pricing curbs alcohol consumption. Stockwell et al. [19] found that a 10% increase in the minimum price of alcohol led to an overall 3.4% decrease in alcohol consumption. Researchers conclude that a minimum pricing policy is far more effective in reducing alcohol consumption and alcohol-related harm than behavioural interventions and campaigns that seek to change behaviour using social marketing and persuasive techniques [10,11,14,18,19].

Despite support from the medical community [20,21] and other advocacy groups [12,22], UK governmental support for the introduction of a minimum price per unit of alcohol was initially “lukewarm” [23] and “less than enthusiastic” [24]. The cited reasons for the lack of support centred about concerns that price legislation may harm the UK alcohol industry, may be in breach of EU competition laws, may unfairly target those on low incomes, and may be perceived to infringe upon the freedom of the general public to drink alcohol however they choose. However, many of these reasons have been dispelled. For example, proponents of the policy acknowledge that the introduction of a minimum price will lead to increased costs for all drinkers, but this will be relatively minimal in moderate drinkers. It is individuals with the highest rates of consumption that are likely to be the most affected financially by an introduction of a minimum price. It is these hazardous drinkers that present the highest risk and are most in need of intervention. There is clear evidence that drinkers with the highest level of consumption gain the vast majority of their alcohol (83%) at prices below the 50p minimum price per unit advocated by the Chief Medical Officer [20,25] and seem to cite expense as a main reason to cut down on drinking [26]. In contrast, moderate drinkers who consume alcohol within-guideline limits would have comparatively smaller increases in price compared to heavier drinkers who regularly consume alcohol above guideline limits [12].

As a consequence of the strong evidence for a minimum price, the UK government has now provided the strongest indication yet that it will introduce a minimum price policy in the near future. Furthermore, the Scottish executive has recently voted to introduce a 50 pence per unit minimum price policy in Scotland [27], which represents a significant milestone after the policy was voted down in 2010 [28]. The policy may yet be subjected to a legal challenge by representatives of the alcohol industry who claim that the policy flaunts European Union competition laws, and such challenges may delay its introduction. The UK government has recently published a revised policy on alcohol which includes proposals to introduce a 40 pence per unit minimum price [29]. The policy also proposes a ban on “below cost” sales of alcohol such that alcoholic beverages cannot be sold at a cost below the sum of government duty and value added (sales) tax for the specific beverage [30]. Although the UK and Scottish governments are among the first to propose the universal introduction of a minimum pricing policy, introductions of partial minimum pricing has been introduced elsewhere. Examples include the ‘alcopops’ tax in Australia and the introduction of a minimum price on spirits in Russia [31]. These have been mooted as having limited success in reducing binge drinking and reinforce the importance of introducing a universal minimum price [32].

In summary, evidence that the introduction of a minimum price per unit will lead to an overall reduction in alcohol consumption in the UK is clear [15] and it appears to be the pricing policy of choice among the majority of organisations and researchers that have examined the effect of pricing on consumption, health, and social outcomes [13,20]. Notwithstanding this general support, there has, to date, been little in-depth investigation of the attitudes and beliefs of the general public toward such a policy.

### The present research

The aim of the present study was to investigate peoples’ attitudes and beliefs concerning the proposed introduction of a minimum price per unit of alcohol in the UK. In addition, the study also aimed to identify the conditions that might increase the acceptability of this policy. Household surveys have consistently shown public support is higher for those policies that provide information or treatment for alcohol abuse than those that raise the price of alcohol or restrict access to alcohol [33-35]. Taking this into consideration, it is reasonable to assume that the general public is likely to be opposed to the introduction of a minimum price per unit of alcohol or to view it with some degree of disfavour. This is despite indications that a minimum price per unit would have a small effect on the household expenditure of moderate alcohol drinkers and a substantially greater effect for harmful drinkers [10]. There is also evidence that the introduction of a minimum price may even save moderate drinkers money as they would not be effectively subsidising the behaviour of harmful and hazardous drinkers who purchase alcohol at the lower end of the retail market that can be heavily discounted due the sales of alcohol in the mid-to-high price range [36]. Nevertheless, there has, to date, been no research on people’s beliefs and attitudes toward such a policy.

Furthermore, no research has sought to identify the possible conditions under which people may be more likely to endorse such a policy. Assuming that people will generally hold negative views towards the policy, we thought it would be important to identify the perceived factors that might make a minimum price policy more acceptable. Such views would be of considerable interest to lobby groups, policymakers, and governments as one of the main reasons behind the UK government’s reluctance to introduce a minimum price policy is that its introduction may have a negative effect on the future electoral performance of the incumbent government given the perceived unpopularity of the policy. The information would be valuable in providing information as to how to pitch messages to the general public regarding a minimum price policy as well as providing information to the few groups in the health professions who have yet to unequivocally endorse the policy [37]. However, such views are speculative given the current gap in knowledge with respect to a minimum price policy, and the current research aims to address this gap.

We conducted a number of focus groups with people from a representative cross-section of community groups to gain in-depth data on their knowledge, attitudes and beliefs with respect to a minimum price per unit policy and, importantly, how it might be made more acceptable. Given indications of modest support for universal legislation to reduce alcohol consumption rather than more targeted strategies, based on research conducted in Australia [38], we anticipated that people would express largely negative attitudes toward the policy and we were interested in whether there were conditions under which they would reconsider their position. The results of these focus groups were expected to provide the first in-depth data on public views toward the minimum price per unit policy and provide information to inform policymakers on means to curb excessive alcohol consumption.

## Methods

### Procedure and interview schedule

Ethical approval was obtained from the Research Ethics Committee in the School of Psychology at the University of Nottingham prior to data collection. Twenty-eight focus groups including a total of 218 participants were conducted to investigate the attitudes and beliefs held by members of the public with respect to the introduction of a minimum price per unit of alcohol policy and investigate the conditions that would maximise the acceptability of the policy. Prior to each focus group, participants were asked to complete the Fast Alcohol Screening Test (FAST) [39] concerning their usual alcohol-drinking patterns. This was to intended provide overall descriptions of each focus group in terms of general levels of drinking with respect to population norms, their typical patterns of drinking, and their overall experience with alcohol. According to Hodgson et al. [40], a score of 3 or more on the FAST questionnaire indicates hazardous drinking. Within the current study 37% (n = 80) of participants were classified as ‘hazardous’ drinkers. Almost half of the hazardous drinkers were University (n = 26) and sixth-form students (n= 12). The remaining ‘hazardous’ drinkers were found amongst the unemployed (n= 10), White collar workers (n= 8), Rural community (n= 7); Hazardous drinkers (n= 6); South Asian (n= 6); Blue collar workers (n= 3), older adults (n = 1) and African-Caribbean community (n= 1). Please see Additional file 1: Appendix 1 for full sample characteristics.

Each focus group was organised according to the same semi-structured interview schedule (see Additional file 1: Appendix 2 for details). The discussions were initiated and led by a facilitator with questions aimed at eliciting opinions and discussion of the minimum pricing policy among participants. Participants were initially introduced to the overall topic of discussion by encouraging brief discussions on alcohol-related topics such as their own drinking behaviour, peoples’ motives for drinking, and the possible antecedents of binge drinking in the UK.^a^ Thereafter the central topic of discussion was the proposed introduction of a minimum price per unit. The policy was explained and participants were provided with clear information as to how it might influence the price of typical alcoholic drinks. Posters were used to help explain how the introduction of a minimum price per unit might affect real alcohol prices. The lowest current price for different brands of lager, cider, wines, and spirits (taken from supermarket price comparison website - http://www.mysupermarket.co.uk) were shown together with the price likely to be set under a minimum pricing policy (i.e., prices were calculated for a minimum price per unit of alcohol set at 40p). The policy was conveyed to participants in a favourable light with the suggestion that the policy “would have the greatest effect on people who typically drink cheap alcohol the most (i.e., young binge drinkers and heavy low-income drinkers)”. As part of the script, participants were also informed of research demonstrating that “an increase in the price of alcohol leads to reductions in consumption, binge drinking, alcohol dependence and the problems associated with these”. Please contact the first author for further details and electronic copies of the posters. Following this, participants were asked to give their opinions about minimum alcohol pricing and the possible outcomes following its implementation, together with a discussion of how the introduction of a minimum price per unit of alcohol might be made more acceptable. The focus groups typically lasted 90 minutes, although the length of groups varied according to group size and the contributions made by participants during the course of the discussions. Participants’ were informed that discussions would be recorded and transcribed in full.

### Participants

In order to reflect views on minimum alcohol pricing likely to be representative of different community groups, focus group participants were recruited from one of the ten target groups selected for the study: (1) sixth-form students (n= 26); (2) university students (n= 41); (3) blue-collar workers (n= 20); (4) white-collar workers (n= 20); (5) unemployed people (n= 19); (6) older adults (n= 44); (7) people from the African-Caribbean community (n= 4); (8) people from the South Asian community (n= 24); (9) people from the rural community (n= 13); and (10) ‘hazardous’ drinkers (n= 7). Each focus group comprised 4 to 16 participants and all were recruited from the same target group. In total, 218 participants took part across the 28 focus groups. Twenty-two focus groups were conducted in the East Midlands region of the UK and a further six were conducted in the North West region of the UK.

### Analytic approach

As there is no previous research investigating people’s views of introducing a minimum price per unit alcohol pricing policy, we adopted an inductive, qualitative approach to identify the prevalent beliefs generated by the sample regarding the minimum price policy. This approach has been advocated for investigations in fields where there is virtually no previous data or information. We used inductive thematic content analysis to identify the themes that emerged from the focus group discussions [41]. Using qualitative data analysis software (NVIVO), themes were identified through multiple readings of the focus group transcripts, cross-checking for common patterns and emergent themes until theme saturation occurred. Consistent with an inductive approach, there was an attempt to be ‘open’ to the data in terms of emerging themes. However, it is recognised that themes interpreted as a ‘tabula rasa’ [42]. It is, therefore, acknowledged that the interpretation of data will be influenced by the researcher’s prior knowledge and views (in this case, by the knowledge and awareness of the researcher regarding the effectiveness of pricing policy in curbing alcohol consumption) but, at the same time, there is an attempt to be open to new findings that may, for example, conflict with existing research or the researchers’ perspectives.

The extent to which the current data generalise to the wider population was not the focus of the present research, instead it aimed to provide detailed data on views and perceptions of the minimum pricing policy. However, we anticipate that the emergent themes from the focus groups will likely transfer to the wider population given our efforts to recruit participants from a diverse range of groups from the UK population. With respect to the transferability of the current findings, we believe that it is the responsibility of the readership to decide the extent to which findings relate to their own experiences and transfer to other groups and contexts. We believe that transferability is enhanced by the provision of detailed descriptions in our analysis (e.g., sufficient lengthy quotes; clear details on process of data collection and analysis) to allow readers a proper understanding of the context and emergent themes. Investigators also have a responsibility to provide sufficient contextual information about fieldwork sites to enable the reader to make such a transfer. Consistent with Davis’ [43] review of qualitative methods, “the responsibility of the original investigators ends in providing sufficient descriptive data to make similarity judgements possible” (p. 606).

## Results

The analysis provided important insight into participants’ attitudes and beliefs with respect to a minimum price per unit of alcohol policy and a number of key themes emerged. In terms of general preliminary findings, it was clear that there was little evidence to suggest that people would unconditionally support the introduction of a minimum price per unit of alcohol. There was an overall opposition to the policy and a general scepticism concerning its effectiveness. Analysis revealed that objections to a minimum pricing policy had three main elements: (1) participants were sceptical of minimum pricing as an effective means to reduce alcohol consumption; (2) participants disliked the policy for a number of reasons - the most frequently cited reason was that the policy unfairly punished those who drink in moderation or ‘sensible’ drinkers; and (3) concerns were also expressed that a minimum price per unit might create or exacerbate other existing social problems. Evidence for these three themes was prevalent in the discussions of all ten of the target groups. For additional quotations derived from the transcripts representing the emergent themes please see Additional file 1: Appendix 3.

### Will a minimum price per unit be effective in reducing alcohol consumption?

**Figure 1:**
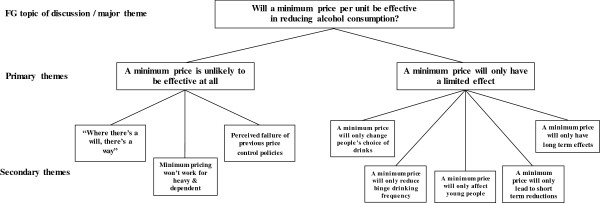
Major theme 1 with associated primary and secondary themes.

The first major theme was scepticism concerning the efficacy of a minimum pricing policy for alcohol to reduce harmful alcohol consumption. Such scepticism was based on: (1) a belief that the introduction of a minimum price per unit would be unlikely to be effective at all and would have no significant influence on UK alcohol consumption, and people would continue to drink regardless of any price increase; and (2) minimum pricing would only have a limited effect on people’s drinking habits, and these effects would, at best, be confined to underage drinkers and subject to limitations (Figure 1).

### A minimum price is unlikely to be effective at all

There was a perception that the introduction of a minimum price per unit policy would be unlikely to have any real impact on alcohol consumption in the UK. This scepticism seemed to stem from one or more of three sub-themes that frequently emerged in focus group discussions: (i) people will drink alcohol if they want to, and would find ways to continue drinking (i.e., “where there’s a will, there’s a way”); (ii) a minimum price would have no impact on the alcohol intake of heavy or dependent drinkers; and (iii) the perceived failure of previous price-control policies.

### Where there’s a will, there’s a way

Participants repeatedly expressed a view that the introduction of a minimum price per unit would have no effect on alcohol consumption, and that people were likely to continue to drink regardless of the proposed price increases. Indeed, participants frequently argued that, if sufficiently motivated, people would find ways to continue drinking following the introduction of a minimum price per unit: “*I think you’d just find another way of getting cheap alcohol… there will always be a way to get cheap stuff I should think.”* [FG6 – University student]. This perception was apparent across the variety of backgrounds (e.g., “*If you want something you’re going to get it aren’t you? Regardless of what the price is, if you want that you’re going to get it*” [FG23- African-Caribbean]) and ages (e.g., “*If they want to drink, they’ll find a way to do it. So personally I don’t think it’ll make much difference at all”.* [FG22 – Older adult]).

Some participants thought that minimum alcohol pricing would lead to significant increases in home brewing, bulk buying, and an influx of cheap foreign imports. In most cases, however, participants simply expected that people would continue drinking, prioritise their spending on alcohol and cut down on expenses elsewhere to maintain their drinking habits: “*I think people will just cut back on other things if they can’t afford to drink*” [FG2- Sixth-form student].

### Minimum pricing won’t work for heavy and dependent drinkers

The second reason for the scepticism was that participants believed that the proposed policy would fail to influence the drinking habits of those who were heavy drinkers or alcohol dependent: *“I don’t think it’ll work on the people who have a drink problem because they will find a way to do it.”* [FG11 – Blue-collar worker]

I think the people who are at the extreme end of the problem, who are the heavy drinkers, who perhaps even drink so much that they’re going to get ill or that they suffer from alcoholism, it doesn’t matter how much it costs, they’ll just find a different way of getting the money to pay for it.

[FG13 – Office worker]

### Perceived failure of previous price control policies

The third reason underlying the scepticism about the effectiveness of a minimum price was, in part, based on the belief that previous price control policies had largely failed to achieve significant changes in the health behaviour of the general public. For example, the perceived failure of increasing taxation to reduce levels of smoking: *“I doubt that it would work for the same reason that smoking continues, and I think possibly drinking is more accepted than smoking. So it stands less of a chance*” [FG12- Office worker]. The belief that price would not alter drinking habits was found across the age spectrum: “*I think they will [keep buying alcohol] whatever price it is, they will buy it. It’s like when cigarettes kept going up and up, they still kept buying them… it didn’t stop them”* [FG20 – Older adult].

### A minimum price will only have a limited effect

Where participants were willing to consider that a minimum price per unit alcohol might reduce alcohol consumption, they were sceptical about the scale of the impact the policy might have and believed it was likely to have only minimal effects. Specifically, participants perceived that the effects of a minimum price on alcohol consumption might be limited in one of five ways: (i) people’s choice of drinks will change; and (ii) people would engage in binge drinking less frequently. Moreover, participants believed that the effects of any reductions would be subject to limitations: (iii) reductions would be confined to particular groups such as young people and underage drinkers; (iv) reductions in alcohol consumption would last a relatively short period of time, after which people would adjust to the higher alcohol prices; and (v) reductions in alcohol consumption would be achieved only after a long period of time, where higher alcohol prices were thought most likely to have the greatest effect on the next generation of drinkers. In each case, it was clear that participants did not regard these incremental changes in drinking behaviour to be significant enough to merit the introduction of a minimum pricing policy: “*It might have a small impact but probably not worth all the effort that they’ll go through to put it through”* [FG7 – University student]. Several believed that the proposed policy would have only a limited impact: “*It could work, have a slight impact, but I don’t think anything like what they would want…It wouldn’t solve the problem”* [FG12 – Office worker].

### Minimum price will only change peoples’ choice of drinks

Many participants put forward the view that the most likely outcome of a minimum pricing policy would be changes in what people drink, not the amount they drink:

*Instead of buying the cheap strong beers, they’ll move on to something like a bottle of scotch, or something which is, hasn’t changed by that much [in price]… So I think it’ll just transfer the problem from people drinking cheap cider, from drinking cheap scotch, and then they’ll be having the same kind of debates in ten years about putting the price of scotch up and it’ll kind of go on from there* [FG8 – University student].

The belief that individuals would react by simply changing the type of drink, for a cheaper alternative was echoed by several participants: “*I don’t think it would solve the problem because these people [binge drinkers]…. would then go to the cheaper one and they buy more because it’s cheaper”* [FG19 – Older adult].

### A minimum pricing policy will only reduce binge drinking frequency

Participants also expected that a minimum price per unit policy, if implemented, may lead people to change their drinking habits. Most significantly, participants were willing to accept that minimum alcohol pricing might lead individuals to engage in binge drinking less frequently. Despite this, participants believed minimum pricing might not lead to an appreciable reduction in overall alcohol consumption in the UK, and may only serve to focus people’s drinking habits more on the more risky heavy episodic ‘binge’ drinking at the expense of safer, more ‘sensible’, moderate drinking: *“[People will] probably drink less often, but more heavily when they do drink”* [FG2 – Sixth-form student] and “*You’re more likely to binge because they can’t do it as often as what they used to…more likely to binge and binge worse than what they used to*” [FG15- Unemployed].

### A minimum price will only affect young people

A commonly held opinion was that any reductions in alcohol consumption as a result of the policy would be confined to young or underage drinkers: “*I think it might only work for young people though…teenagers prefer to drink the cheaper stuff, so it’ll only affect them really* ”[FG3 – Sixth-form student] and *“I think it’s only going to affect people who are under eighteen, who are trying to drink as much as they can but for as cheap as possible”* [FG27 – Rural community].

### A minimum price will only lead to short term reductions

Participants also expressed the view that a minimum price policy was likely to produce only short term reductions in alcohol consumption in the UK, believing that after a certain period of time alcohol drinkers would adjust to the higher prices and return to their previous pattern of drinking: “*My personal use would probably go down, and then probably go back up again [after I] got used to the price change*” [FG1 – Sixth-form student]. This view was also expressed by an office worker:

*To be honest, I don’t think it’s going to affect anyone in the long term. Like, initially they’ll be an outcry, and people will be like oh I can’t afford white lightening and then they’ll be a shift, and they’ll be exactly the same. I don’t think it’s going to affect anyone long term at all* [FG13].

### A minimum price will only have long term effects

Despite the general scepticism, participants believed that higher alcohol prices were more likely to affect future generations of drinkers, and the policy would have no immediate effect on the problems of excess alcohol consumption in the current population:

I think it’ll work. I think the impact on the generations now, would be minimal to average. I think going forwards, [for the] next generation and the ones after that, I think it’ll just become accepted and that’s how it is, and I think that’ll be the biggest benefit.

[FG11 – Blue-collar worker]

Several participants did not believe a minimum price per unit would significantly influence the drinking habits of those who are already consuming alcohol, and change might only be possible for future generations unaccustomed to drinking: “*I don’t think it’ll have an amazing difference…it will have an effect, an eventual effect, not an immediate effect*” [FG26- South Asian].

### Do people like the minimum price per unit of alcohol proposal?

**Figure 2:**
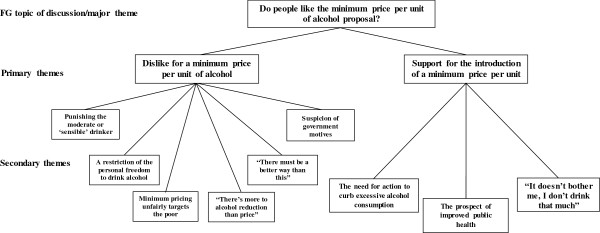
Major theme 2 with associated primary and secondary themes.

Participants were found to dislike the idea of minimum alcohol pricing, for one or more of six main reasons (see Figure 2 for an overview): (i) a minimum price per unit would indiscriminately target all drinkers, unfairly ‘punishing’ those who drink in moderation or ‘sensible’ drinkers at the expense of those drinking in excess of guidelines limits; (ii) minimum pricing was perceived as a restriction on people’s personal freedom to drink alcohol however they chose; (iii) a minimum price was also considered unfair because it would disproportionately affect the lives of the poor more than the rich; (iv) a minimum price was considered a ‘reductionist’ intervention, and would not address the complex interaction of psychological, social, and cultural issues participants believed to be responsible for growing alcohol consumption in the UK (i.e., “there’s more to alcohol than price”); (v) participants believed there were other, more effective ways to reduce consumption other than increasing the price of alcohol that were being overlooked the UK government (i.e., “there must be a better way than this”); and (vi) participants suspected the UK government were likely to have ulterior political motives for the introduction of a minimum price aside from the improvement of public health.

#### Punishing the moderate or ‘sensible’ drinker

This perceived failure to appropriately target the behaviour of a minority of problem drinkers was one of the main reasons we identified for the dislike of the policy. Indeed, participants’ responses frequently reflected the view that minimum pricing was a universal rather than targeted approach to pricing that would serve to unfairly punish those who drink sensibly, at the expense of the more ‘reckless’ behaviour of binge drinkers:

*[It] seems to be punishing people… people [that] haven’t done anything wrong, there’s some people who are going out and getting absolutely drunk, and ending up in hospital, and that is a strain on our society. But a lot of people are just drinking socially and having a good time, we’re not posing a problem to our country at all. So I don’t see why we should be punished by high prices* [FG6 – University student].

Personally I feel a bit offended that it would be people like myself that’ll be having to pay some more money… I just feel the principal of it is unjust, when it’s sensible drinkers who are not causing the problem, but they’re still going to end up having to pay for it.

[FG13 – Office worker]

The theme of punishment and unfairness on moderate and sensible drinkers was echoed across the sample: “*I’m a responsible drinker, why should I get penalised for people that can’t control themselves*” [FG11- Blue-collar worker] and is consistent with previous research showing that the general public favour targeted rather than universal controls to curb alcohol consumption [38].

### A restriction of personal freedom to drink alcohol

A number of participants believed that higher alcohol prices would, to some extent, restrict an individual’s freedom to drink however they chose. In this context, it was evident that participants regarded the introduction of a minimum price as an unwelcome regulation of drinking behaviour by the state, and detracted from the autonomy of individuals to determine their own alcohol intake: “*I don’t agree with it. I don’t see [why] the government should tell us what we can do, and what we can’t do”* [FG11 – Blue-collar worker]. Many participants regarded the introduction of a minimum price as an overly prescriptive approach, where the increase in the price of cheap alcohol would, in effect, force drinkers to change their drinking habits rather than leaving individuals to reduce their alcohol intake of their own volition:

*I think we live in too much of a nanny culture as it is… there are too many rules and regulations about what to do, what not to do. If people want to slowly kill themselves because of alcohol, [they can]. Yes, it has a huge burden on the NHS and therefore society as taxes as a whole, but at the end of the day this isn’t necessarily going to stop them… I think people should be left alone* [FG12 – Office worker]

### Minimum pricing unfairly targets the poor

Some criticisms of the policy were that it would unfairly reduce the quality of life for drinkers from disadvantaged backgrounds, whilst the lives of the financially more privileged would remain largely unaffected and continue to drink however they chose. This perceived inequality was considered by many participants to be unfair and perhaps even socially divisive:

*But there are people on low incomes, if they want to go and have a drink that’s going to cost them a lot of money. They’re going to be less well off, and they probably will still drink to the same extent, and will have less money, maybe for their children or whatever* [FG9 – University student]

Participants felt that such pricing policies would place a disproportionately higher financial burden on those from disadvantaged backgrounds and as such would be unfair: “*The only sort of downside for me is that people on low-incomes, they should be able to a drink, and they shouldn’t be excessively penalised because of this*” [FG12 – Office worker] and “*It targets low-income people basically…so in that sense…it’s not fair*” [FG14- Office worker].

### There’s more to alcohol reduction than price

Participants frequently expressed the view that focussing solely on the relationship between price of alcohol and consumption was simplistic, and did not appropriately reflect the multidimensional nature of the problem: “*I’m not really in favour of it because it doesn’t address the core issues that make people drink in the first place”* [FG3 – Sixth-form student].

Instead, participants often observed that alcohol consumption was the result of a complex interaction of several social and cultural factors, and criticised the idea of a minimum price for its failure to recognise this complexity and to intervene accordingly:

*It’s almost a cultural thing, and we’re trying to put a sticky plaster on it… by doing the price thing, and it’s a deeper thing than that. And it is going to penalise the people who like a drink, and it’s not a problem for them* [FG12 – Office worker].

It was evident that many participants considered the policy ‘reductionist’, and, as such, would fail to address the root causes of excessive alcohol consumption in the UK: “*It’s not addressing the proper issue is it? Why do people drink too much? It’s not answering that*” [FG13- Office worker].

### There must be a better way than this

Participants believed there were better ways to address excessive alcohol consumption in the UK. Accordingly, disapproval of a minimum price was, in some cases, based simply on the idea that alternative interventions had been overlooked: *“Well the government seem to think that is the answer to everything… putting prices of things up. They don’t look at other ways round it*” [FG19 – Older adult] and “*I’m not in favour, because I think there’s other ways of dealing with the problem*” [FG20 – Older adult].

Research evidence has indicated that many of the interventions that participants advocated most strongly (e.g., educational programmes, mass-media campaigns) have, for the most part, had modest effects on reducing alcohol consumption and alcohol-related harm [44,45].

### Suspicion of government motives for introducing a minimum price

Many participants believed that a minimum pricing policy would serve the interests of the government by generating additional tax revenues and serving to garner favour with the general public by convincing them that steps were being taken to tackle the problems associated with excess alcohol consumption. In both cases, it was clear that many participants believed the UK government was likely to have an ulterior motive for the introduction of a minimum price: *“I’m not in favour, because it’s a sneaky way of getting more money out of people for the treasury”* [FG13 – Office worker]. Another refers to the proposed policy as: *“it’s a tax in everything but name”* [FG22- Older adult].

### Support for the introduction of a minimum price per unit

There was also support for means to address excess alcohol consumption. Accordingly, participants who supported or, at least, did not object to the introduction of a minimum price did so for one or more of three reasons (see Figure 2 for an overview): (i) the need for action to curb excessive alcohol consumption; (ii) the prospect of improved public health, particularly among young and underage drinkers; and (iii) the perception that the policy would not have a significant effect on participants’ personal drinking habits (i.e., “it doesn’t bother me, I don’t drink that much”).

### The need for action to curb excessive alcohol consumption

Focus group discussions showed that some participants were in favour of action to address the growing problems of excess alcohol consumption in the UK: “*I think it’s quite a big problem, and something needs to be done to sort it out”* [FG2 – Sixth-form student] and “*But if they go ahead with it, I don’t think I’d complain because I know they’ve got to do something”* [FG11 – Blue-collar worker].

### The prospect of improved public health – a price worth paying

In many cases, participants seemed willing to overlook their own personal objections to a minimum price per unit, and expressed support for its introduction given the prospect of significant public health improvements that the policy might bring. In particular, the idea that a minimum price was likely to reduce the alcohol intake of underage drinkers and improve health outcomes for future generations seemed especially persuasive to participants:

*I’m in favour of it… For the simple reason you’ve got to think of the younger generation. I’m not bothered about the alcoholics, they’ve been drinking for years and years. It’s the younger generation that I think we’ve got to educate… and if by putting it up a few pence stops even one of them buying it and drinking it’s worth it* [FG11 – Blue-collar worker]

“*I am in favour of it…stopping underage drinking and obviously the people that are alcoholics to reduce their consumption*” [FG28- Hazardous drinkers]

### It doesn’t bother me, I don’t drink that much

Some held indifferent attitudes towards the policy and this appeared to be because as individuals, they were not regular or heavy drinkers: “*I’m in favour of it because I’m not a drinker*” [FG- Unemployed]. Another participant said: *“I’m not too bothered to be honest, because I don’t really drink much and I don’t really care what everybody else does”* [FG25 – South Asian].

### A minimum price policy might make matters worse – unintended consequences

**Figure 3:**
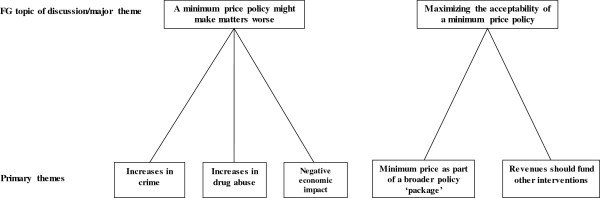
Major themes 2 and 3 with associated primary themes.

In addition to believing that minimum pricing would not work, a frequently-occurring theme was the belief that a minimum price per unit policy might also create or exacerbate other social problems. In particular, participants considered a minimum price would make things worse in one or more of three ways: (i) crime was likely to increase because some drinkers might not be able to afford the higher alcohol prices; (ii) drug abuse was likely to increase because the increasing the price of alcohol would lead drinkers to seek out cheaper alternatives; and (iii) higher alcohol prices would also have negative economic impact (see Figure 3 for an overview).

### Increases in crime

The foremost concern expressed by participants about the minimum price per unit policy was that its introduction might lead to increased levels of crime. Specifically, participants expected that because some drinkers, in particular those who are alcohol dependent, might not be able to afford the higher alcohol prices, they may turn to crime to continue drinking:

*There will not be any long-term benefit… because it’ll cause problems elsewhere. People [who] need a drink, they’ll get it by other sources. If they can’t afford it they’ll go out and steal, whether it’s the wines and spirits themselves, or steal money, or rob, they’ll do whatever* [FG10 – Blue-collar worker].

Participants appeared to focus on the effects a minimum price per unit policy might have on the behaviour of heavy or dependent drinkers and the social consequences: “*Crime will go up, definitely… because I think people who are alcoholics, they need to drink…if you put the price up, they need the money, the crime will go up”* [FG24 – South Asian].

### Increases in drug abuse

Participants were also concerned that the introduction of a minimum price per unit might lead to an increase in drug abuse. Specifically, it was anticipated that increasing the price of cheap alcohol would force some drinkers, especially those who are alcohol dependent, to turn to drugs as a cheaper alternative to serve their needs: “*It’s working out now that a line of cocaine is cheaper than a pint of beer from a pub… So [people] might go on to some other form of drugs instead because it would be cheaper*” [FG1 – Sixth-form student]. There was a perception that individuals might switch from alcohol to illegal drugs: “*Well they’ll start taking cocaine because cocaine will be cheaper than buying alcohol so then you’ve got a cocaine problem*” [FG19- Older adult].

### Negative economic impact

Participants were concerned that introducing a minimum price might lead to job losses and reduced profits for the alcohol industry: “*[It] would probably reduce revenues for the bars and stuff, which would be a kind of big disadvantage against this”* [FG4 – University student]. Others were concerned about the potential for increased levels of indebtedness among certain groups of drinkers:

*I think if anything, it’ll just cause financial problems because a lot of the people who drink heavily, for example, people who have no houses, students, and like people who are unemployed maybe, are stressed so they drink a lot. They haven’t got any money anyway, and they’re still doing it. So students will just get more overdrafts, more loans and get in more debt, as will everyone else who’s got no money and is still drinking…it’ll just cause more financial difficulties* [FG13- Office worker].

Some participants regarded the introduction of a minimum price per unit as a potential obstacle to economic growth, and likely to create unnecessary financial difficulties for those from disadvantaged backgrounds.

### Maximising the acceptability of a minimum pricing policy

When asked how the introduction of a minimum price might be made more acceptable, two themes emerged from the analysis: participants suggested that (i) a minimum price per unit should be introduced as part of a broader package of government policies to address excessive alcohol consumption; and (ii) revenue generated by higher alcohol prices should be used to fund other interventions. In both cases, it was evident that participants were more likely to accept a minimum pricing policy if it was introduced together with other government policies.

### A minimum price as part of a broader policy ‘package’

It was evident that the policy would be more acceptable to many participants if introduced alongside educational programmes and the greater provision of public health information for drinkers (e.g., health warnings, advertising, clearer labelling):*“I think I would be in favour of it, but not on its own. I think it’s one tool in the armoury so to speak”* [FG12 – Office worker]. An educational approach to alcohol consumption was also deemed important: *“If this is combined with other schemes then it will work very well…help in education, like teaching young children about alcohol, even units thing at a young age, then the programme will work but otherwise on its own it won’t”* [FG2- Sixth-form student].

The need for a multi-faceted approach to reduce alcohol consumption was highlighted amongst participants and this included a focus on the health warnings of excess alcohol: “*I think there needs to be a combined approach, like the pictures you get on cigarette packets now, you know obscene throat cancer and stuff like that. It needs to be a joined up approach*” [FG11- Blue collar worker].

### Revenues should fund other interventions

Most participants were receptive to the idea that any additional revenue generated by such a policy might be used to finance the implementation of other alcohol initiatives to reduce excess alcohol consumption in the UK. In this context, participants believed that the proposed policy might be made more acceptable if put forward as a means to fund public services or other intervention policies perceived to be most effective in reducing alcohol consumption: “*I’m against it. I don’t think it’ll work, and I’d probably be more for it if I thought the profits were going to go to the NHS, and the police”* [FG1 – Sixth-form student]. Many participants were against the policy but may be more accepting of it if they could be reassured that any revenue generated from higher alcohol prices would be used to fund other strategies to help tackle the problems associated with excess alcohol consumption:

*I think that this proposal alone wouldn’t work, therefore I’m not in favour of it. But I think there’s a possibility that they could use the extra money they’re making on the alcohol to put directly into other ways of tackling the same issues, and then the two combined could work, but this proposal alone I wouldn’t agree with* [FG13 – Office worker].

One particular quote, appears to pull together several themes from the current study including the important theme regarding why people are drinking to excess: ‘there must be a better way than this’, in addition to ‘suspicion of government motives’ and the importance of introducing ‘a minimum price **only** as part of a broader policy package’:

*I don’t see that’s going to help the situation. I’m also a bit suspicious as to where the money would be going…if it was going in to helping people with alcohol-related issues then fair enough. If it was going into education in school, maybe setting-up community centres and youth facilities so that kids weren’t bored and hanging round the streets, and didn’t feel the need to go out and have two bottles of cider…on a nightly basis, then it’s a good idea*. [FG12- Office worker].

## Discussion

The major theme that emerged in the current study was scepticism regarding the effectiveness of the policy. The overwhelming feeling was the people will continue to drink and will adjust to the higher prices, or change choice of drink. Notwithstanding these perceptions, there is clear evidence that price controls in other areas of public health have been extremely efficacious in changing health behaviour. A prime example is the implementation of price increases on cigarettes which has played an important role in encouraging smokers to stop and has led to reduced smoking both in the UK and countries across Europe [46,47]. This is also coupled with evidence that increasing taxation and price on alcohol in general leads to population-level reductions in alcohol consumption [15,17]. These views identify misconceptions endemic among participants with respect to the economic links between price increases and purchasing behaviour, particularly when it comes to taxation and health-related behaviours like smoking and alcohol cessation. Policymakers interested in introducing a minimum price per unit of alcohol should be aware of this limited understanding and clearly highlight the substantially greater effectiveness of economic interventions such as the proposed minimum price policy in reducing alcohol consumption relative to the modest effects of behavioural campaigns [48,49].

Participants expected most drinkers to respond to the introduction of a minimum price by switching beverages. There are two lines of reasoning here and both represent a possible misunderstanding of the policy. The well-documented argument for switching from a relatively weak alcoholic beverage (e.g., beer) to a much stronger beverage (e.g., whisky) as a response to increases in price [50,51] would not be a strategy to gain cheaper alcohol. This is because the stronger beverage would be at least as expensive due to its high alcohol content and the relative nature of the policy. The argument for switching to the least expensive alternative would also be an ineffective means to gain cheaper alcohol as the least expensive would be weaker in terms of its alcohol content. It seems that participants were acutely aware that price determines beverage choice among drinkers, particularly heavier drinkers, as corroborated in previous research [52]. This suggests that under the policy people would focus on finding drinks that were lowest in price. Under the proposals, these would likely be the weakest in strength and may therefore reduce alcohol consumption. Interestingly, this seemingly modest change in drinking habits was frequently dismissed as inconsequential, and most participants did not believe that these changes in drink choice would lead to population-level decreases in alcohol consumption. It would, therefore, be important to dispel these misunderstandings in educational messages to a sceptical public. In particular, such messages should challenge misconceptions surrounding the effectiveness of the policy. Specifically, messages should highlight that relatively small decreases in alcohol consumption at the individual level is likely to lead to significant aggregate reductions in overall alcohol consumption nationally. There was some recognition that the policy would affect future generations of drinkers but participants felt that there would be no immediate effect.

The second major theme that emerged in the current study was the general dislike of the policy; it was seen by many as unfair and seen as disproportionately affecting disadvantaged groups in addition to punishing sensible drinkers. The policy was considered by many as a reductionist intervention that did not or could not address the complex interaction of psychological, social and cultural issues responsible for the growing alcohol consumption. Participants wanted to see multi-faceted approaches to reduce alcohol consumption and would be more accepting of the policy if they were given reassurance that any revenue generated from higher alcohol prices would be used to fund additional strategies to tackle the problems and causes of excessive alcohol consumption. Therefore, the introduction of a minimum price policy should be accompanied by messages highlighting that a reduction in harmful levels of alcohol consumption, a key target outcome of a minimum pricing policy, will likely lead to a lower incidence of the maladaptive health outcomes and reduced expense to health services attributable to hazardous forms of drinking.

If implemented, a minimum price per unit is expected to have the greatest effect for people who buy discounted alcohol on a regular basis and have patterns of drinking behaviour considered excessive and, as a consequence, harmful, regardless of their age or background [15,53-55]. However, participants did not generally share this expectation; this misconception will need to be addressed if a minimum price is to be endorsed by the public as an effective means to reduce alcohol consumption in the UK.

Although in general, there was scepticism regarding the effectiveness of the policy and cynicism regarding the motive underlying its introduction, there was some support for the policy amongst participants. Many participants recognised a need for action to curb excessive alcohol consumption, particularly in the potential for reduction of alcohol consumption in underage drinkers. For the participants, the policy would be more acceptable if minimum pricing was part of a broader package alongside educational approaches and greater provision of public health information for drinks. Researchers and organisations have also advocated the adoption of multiple intervention strategies at the population (e.g., pricing) and individual (e.g., brief psychosocial interventions) levels [22,56-64]. Such a multi-faceted approach is logical given the evidence supporting the effectiveness of interventions using either of the approaches. Future research should explore whether a minimum pricing policy in combination with other intervention strategies such as mass-media information campaigns will have synergistic effects on alcohol consumption. It is, however, clear from the present study that participants believe that the effectiveness of a minimum price policy will be reduced, and its acceptability impaired, without the introduction of additional measures. Current findings suggest that an optimal strategy to increase the acceptability of a minimum price policy would be to include an educational campaign aimed at changing beliefs and attitudes toward minimum price itself in order to dispel any misconceptions surrounding its effectiveness as a standalone policy [65], in addition to campaigns aimed at increasing public awareness of appropriate and safe drinking levels, and the damaging effects of binge drinking, particularly aimed at youngsters, alongside a minimum price policy.

## Conclusions

The aim of the present study was to investigate peoples’ attitudes and beliefs regarding the introduction of a minimum price per unit of alcohol policy in the UK, and to identify the conditions that might increase the acceptability of this policy. The present research is unique given the relative dearth of information on people’s beliefs and attitudes toward this policy and how they might respond to its introduction. Furthermore, no in-depth investigation has examined the acceptability of this pricing policy and the strategies that individuals perceive might increase its acceptance among the general public. The present findings are therefore expected to inform how policymakers might effectively introduce a policy of alcohol minimum pricing, and maximise its public acceptance. A further strength of the current research is that the data are derived from a large number of focus groups (N = 28) reflecting the views of over 218 participants from a diverse set of community groups.

Overall, the present data suggested that participants’ were largely sceptical of the effectiveness and motives behind the introduction of a minimum price per unit policy for alcohol. Objections to the policy may be attributed to three main issues: (1) a misunderstanding of the minimum price per unit policy itself; (2) the failure to recognise the significance of small incremental reductions in alcohol consumption; and (3) a preoccupation with the effects of a minimum price on heavy and dependent drinkers.

Despite accepting that changes in peoples’ drinking habits were possible, most participants were sceptical that a minimum price per unit was likely to bring about an immediate reduction in alcohol consumption. This scepticism was arguably the result of a misunderstanding of the policy. There was a general failure to recognise that small, seemingly inconsequential, changes in the drinking behaviour at the individual level are likely to equate to substantial reductions in overall alcohol consumption at the population level. For example, despite expecting that a minimum price might encourage drinkers to switch from stronger to weaker alcoholic beverages, many participants did not consider its introduction would lead to substantial reductions in alcohol consumption. Based on these findings, the challenge for policymakers is not only to appropriately manage public expectations as to what a minimum price per unit is likely to achieve, but also to demonstrate how incremental changes in individual behaviour will likely lead to aggregate reductions in alcohol consumption and alcohol-related harm in the UK.

Objections to a minimum price per unit also showed that many participants were preoccupied with the limited effects of a minimum price on the behaviour of heavy and dependent drinkers. Specifically, participants were critical of a minimum price per unit because it was considered unlikely to deter heavy or dependent drinkers from drinking to excess. It would seem that some participants who opposed the introduction of a minimum price assumed that alcohol dependence was the most important public health issue associated with excessive alcohol consumption and that this was the main target of introducing minimum price. While alcohol dependence is a serious health threat, there is a need to challenge the assumption that pricing policy should be directed exclusively at dependent drinkers. It should be highlighted that other hazardous patterns of drinking in which people regularly consume alcohol above guideline limits present a proportionately greater risk to public health issue than alcohol dependence.

An additional aim of the present research was to identify the conditions likely to improve the acceptability of a minimum price per unit policy and how it might be introduced more effectively. Findings indicate that a minimum price per unit would be most acceptable to participants if introduced together with additional policies (e.g., media campaigns highlighting the harmful effects of alcohol) also aimed at reducing excessive alcohol consumption. It was evident that participants regarded the synergistic effects of such a policy mix was most likely to achieve significant reductions in alcohol consumption and alcohol-related harm, and in so doing it would serve to address the foremost objection, namely that a minimum price per unit would not work effectively in isolation. The introduction of a minimum price per unit would be more acceptable to the public if introduced as the centrepiece policy as part of a wider UK government strategy to curb excess alcohol consumption. However, given the limited levels of understanding demonstrated by participants of the policy, the most efficacious approach to promoting acceptance of a minimum price per unit would be to provide sufficient and appropriate information to dispel the negative beliefs that the policy will have limited effects, highlight that the policy will likely lead to population-level reductions in alcohol, and make clear that the policy would have a much lower effect on the pockets of moderate drinkers relative to heavier drinkers and those who consume alcohol to excess [54].

### Limitations and future research

The present findings suggest the most significant barrier to public support for a minimum price per unit is likely to be people’s scepticism regarding the effectiveness of the policy to significantly reduce harmful alcohol consumption. Future research should therefore aim to seek to identify education interventions that improve public opinion most effectively and serve to allay misconceptions regarding a minimum price; such an intervention is likely to prove useful as a means to facilitate the introduction of the policy.

Although the present findings provide an important first insight into public opinions about the introduction of a minimum price per unit of alcohol, the present study had a number of limitations that should be acknowledged. Caution should be exercised when generalising from focus group data alone. Using focus groups, the present study aimed to provide rich, detailed data on peoples’ beliefs regarding the minimum pricing policy. We collected data from a large number of focus groups from diverse populations in order to canvass views and obtain sufficient coverage of public opinion on minimum price from numerous important groups within the population, with the potential for *transferability* of the emerging themes to other groups. However, we recognise that we are not able to comment on the extent to which findings may be true of people in other settings. Conducting similar research in different settings would be valuable and further contribute to converging knowledge regarding perceptions of minimum pricing. The use of focus groups in the current study did not allow us to make definitive comparisons to establish whether attitudes and beliefs concerning a minimum price varied significantly between the different communities investigated. Accordingly, future research should further investigate the main themes raised in this investigation and explore the possibility that people from different community groups might hold differing views about the introduction of a minimum price per unit. The current study is the first to explore attitudes towards minimum pricing and offers a baseline understanding of the general public’s perceptions of a minimum price policy with which findings of subsequent research should be compared. Future qualitative research exploring public opinion should also provide further details to participants such as evidence and statistics about who spends what on alcohol, evidence that prices in the on-trade would be largely unaffected, and a range of example prices for each beverage.

## Endnote

^a^ It must be acknowledged that binge drinkers have been cited as largely consuming alcohol from on-trade establishments were alcohol prices are likely to be above proposed minimum price thresholds, suggesting that minimum pricing may have less effect on binge drinking than previously mooted [29]. Sheffield modelling data supports this indicating that introducing a minimum price will have less effect on hazardous drinkers aged 18-24 compared with other age groups [10]. However, these data may not account for the increasing trends in drinking alcohol purchased from the off-trade by young binge drinkers prior to visiting on-trade establishments, a practice known as ‘pre-loading’ or ‘pre-partying’ [66], so minimum pricing may also affect these types of drinkers.

## Competing interests

The authors declare they have no competing interests.

## Authors’ contributions

AJL developed the methods including the interview schedule, recruited participants and conducted the focus groups, transcribed the data, conducted the analysis and extracted the themes, conducted the literature review, and drafted the manuscript. SJH helped with the analysis of focus group data and with the writing of the methodology, results and discussion. MSH conceived the study, secured the funding to conduct the research project, designed the methods and designated the approach, directed the data analysis, and drafted and commented upon sections of the manuscript. All authors read and approved the final manuscript.

## Pre-publication history

The pre-publication history for this paper can be accessed here:

http://www.biomedcentral.com/1471-2458/12/1023/prepub

## Supplementary Material

Additional file 1 **Appendix 1** - Participant Screening and Characteristics. **Appendix 2** - Focus Group Interview Schedule. **Appendix 3** – Additional quotations. Click here for file
