# Supplementary material for: A minimum price per unit of alcohol: A focus group study to investigate public opinion concerning UK government proposals to introduce new price controls to curb alcohol consumption
Source: BMC Public Health. 2012 Nov 23;12:1023. doi: 10.1186/1471-2458-12-1023 (PMC3740777; doi:10.1186/1471-2458-12-1023)
Supplement: Additional file 1 — Appendix 1 - Participant Screening and Characteristics. Appendix 2 - Focus Group Interview Schedule. Appendix 3 – Additional quotations. [file 1471-2458-12-1023-S1.doc]

## Additional Files

## Additional file 1: Appendix 1 - Participant Screening and Characteristics

(To be Made Available as Supplemental Material Only)

## Participant Alcohol Consumption

Before each focus group, participants were asked to complete a brief questionnaire concerning their usual alcohol-drinking patterns. Participants were asked how many units of alcohol they had consumed in the previous two weeks and how many occasions the consumed 10 units or more for men, or seven units or more for women, on a single drinking occasion over the previous two weeks. Drinking in excess of these limits was used as a measure of the number of times participants engaged in binge drinking in the two week period prior to the focus group. In addition to this, participants completed the four-item Fast Alcohol Screening Test (FAST) to assess the extent of participants’ alcohol misuse. This instrument has rigorously evaluated and demonstrated validity and reliability as a brief means to evaluate the extent of heavy drinking . A FAST score of 3 or above is regarded to indicate that a participant is a ‘hazardous’ drinker.

## Sixth-form students

Twenty-six sixth-form students (21 male, 5 female, mean age = 16.85, *SD* = 0.37) took part in three focus groups (Focus group 1 (FG1), *n* = 6; FG2, *n* = 10; FG3, *n* = 10). On average, participants consumed 5.96 units of alcohol (*SD* = 6.09) and reported binge drinking 0.35 times (*SD* = 0.69) in the two weeks prior to the focus group. FAST scores indicated that 12 of the 26 participants could be classified ‘hazardous’ drinkers.

## University students

Forty-one university students (18 male, 23 female, mean age = 20.88, *SD* =3.23) participated in six focus groups (FG4, *n* = 8; FG5, *n* = 6; FG6, *n*= 8; FG7, *n* = 6; FG8, *n* = 7; FG9, *n* = 6). On average, participants reported consuming 21.63 units of alcohol (*SD* = 28.53) and binge drinking on 1.20 occasions (*SD* = 1.93) in the two weeks prior to the focus group. FAST scores indicated that 26 of the 41 participants could be classified as ‘hazardous’ drinkers

## Blue-collar workers

Twenty employees of a national supermarket chain (4 male, 16 female, mean age = 47.40, *SD* = 9.82) took part in two focus groups (FG10, *n* = 5; FG11, *n* = 15). Participants were reported consuming 17.05 units of alcohol (*SD* = 18.01) and binge drinking 0.35 times (*SD* = 0.75) in the prior two weeks. FAST scores indicated that 3 of the 20 participants could be classified as ‘hazardous’ drinkers.

## White-collar workers

Twenty administrative office workers from the University of Nottingham (5 male, 15 female, mean age = 34.80, *SD* = 9.36) took part in three focus groups (FG12, *n* = 6; FG13, *n* = 8; FG14, *n* = 6). Participants consumed 18.55 units of alcohol (*SD* = 15.88) and reported binge drinking 0.55 times (*SD* = 0.76) in the two weeks prior to the focus group. FAST scores indicated that 8 of the 20 participants could be classified as ‘hazardous’ drinkers.

## Unemployed

Nineteen adults who were unemployed at the time of the study (13 male, 6 female, mean age = 36.26, *SD* = 12.56) participated in three focus groups (FG15, *n* = 7; FG16, *n* = 4; FG17, *n* = 8). They consumed 29.79 units of alcohol (*SD* = 36.08) and reported binge drinking 1.37 times (*SD* = 2.24) in the two weeks prior to the focus group. FAST scores indicated that 10 of the 19 participants could be classified as ‘hazardous’ drinkers.

## Older Adults

Forty-four older adults (23 male, 21 female, mean age = 69.27, *SD* = 8.96) took part in five focus groups (FG18, *n* = 6; FG19, *n* = 9; FG20, *n* = 5; FG21, *n* = 8; FG22, *n* = 16). Participants reported consuming 12.00 units of alcohol (*SD* = 12.95) and binge drinking 0.24times (*SD* = 1.03) in the previous two weeks. FAST scores indicated that one of the 44 participants could be considered a ‘hazardous’ drinker.

## African-Caribbean

Four people who described their ethnicity as African-Caribbean (2 male, 2 female, mean age = 54.00, *SD* = 19.20) took part in a single focus group (FG23). They consumed 4.00 units of alcohol (*SD* = 1.83). In the two weeks prior to the focus group, and over the same period, none of them reported binge drinking. FAST scores indicated that one of the four participants could be considered a ‘hazardous’ drinker.

## South Asian

Twenty four people who described their ethnicity as either Asian-Indian or Asian Pakistani (16 male, 8 female, mean age = 40.29, *SD* = 20.33) took part in three focus groups (FG24, *n* = 9; FG25, *n* = 7; FG26, *n* = 8). Participants reported consuming 6.33 units of alcohol (*SD* = 12.85) and binge drinking 0.13 times (*SD* = 0.45) in the two weeks prior to the focus group. FAST scores indicated that 6 of the 24 participants could be classified as ‘hazardous’ drinkers.

## Rural Community

Thirteen participants (4 male, 9 female, mean age = 21.82, *SD* = 3.37) took part in a focus group (FG27) held in West Cumbria in the North West region of England. Participants described themselves as White-British and reported consuming25.67 units of alcohol (*SD* = 32.33) and binge drinking 1.42 times (*SD* = 1.16) in the two weeks prior to the focus group. FAST scores indicated that 7 of the 13 participants could be classified as ‘hazardous’ drinkers.

## Hazardous Drinkers

Seven participants (4 male, 3 female, mean age = 20.71, *SD* = 2.29) took part in a focus group (FG28) held in Lancashire in the North West region of England. When asked to indicate their ethnicity, six participants described themselves as White-British and one as of ‘other ethnic background’. Participants consumed 57.71 units of alcohol (*SD* = 38.11) and reported binge drinking 3.00 times (*SD* = 1.73) in the two weeks prior to the focus group. FAST scores indicated that 6 of the 7 participants could be classified as ‘hazardous’ drinkers.

## Additional file 2: Appendix 2 - Focus Group Interview Schedule

(To be Made Available as Supplemental Material Only)

**Drinking behaviour**

What is you ‘normal’ drinking behaviour? Talk me through an evening from the start?

***Where*** do you normally drink? - What factors influence *where* you drink?

***What*** do you normally drink? - What factors influence *what* you drink?

***When*** do you drink? - What factors influence *when* you drink?

Do you ever set out with the explicit purpose to get drunk? **– If so, why?**

Do you ever drink within the recommended guidelines on a night out? (2-3 units for women, 3-4 units for men) - **If so, why? If not, why not?**

Have you ever considered a night out with your friends where you don’t drink at all? **If not, why not?**

**Motives for drinking**

Why do you drink?

In what situations do you find yourself drinking the most?

What do you think might trigger you to drink more than you normally do?

What factors do you think might limit the amount you would drink on a night out?

**Increase of alcohol-related problems**

The latest research and news reports both suggest people are drinking more alcohol, and binge drinking more frequently, leading to greater incidence of alcohol-related problems (i.e. crime, hospital admissions, and social disorder).

**Why** do you think people are drinking more?

**What** do you think is responsible for the increase in binge drinking?

- Change to opening hours?
- Alcohol advertising?
- Supermarket discounts? / Drinks promotions?

Do you think these problems might be caused by the increased affordability of alcohol?

**Minimum price per unit – A brief explanation**

One reason why alcohol consumption has increased over the last 50 years is thought to be the **increasing affordability of alcohol**.

To address the problems associated with increasing alcohol consumption, and binge drinking in particular, the UK government is considering introducing a **minimum price per unit of alcohol**.

The Scottish parliament already have plans to change how alcohol is priced, **where each unit of alcohol in a drink will have a minimum price of around 40 – 50p**. This policy might now be introduced nationwide for the UK as a whole.

The price-per-unit prices shown on the posters are calculated based on the assumption that a unit of alcohol is given a minimum price of 40p (i.e. prices would obviously be much higher if based on 50p per unit). So as you can see, **the more alcoholic a drink is**, **the higher the minimum price** is likely to be sold for in the supermarket / off-licence.

Minimum pricing would simply mean that all alcohol would have a minimum price under which alcohol could not be sold, although retailers would be free to charge above this. For example, the price of brand name alcohols will not change significantly because they often cost more than the ‘minimum price’ guideline. This minimum price would be set according to the number of alcoholic units a drink contains, not the percentage strength of a drink.

If implemented, this minimum price unit policy is expected to significantly increase the price of those drinks that are already very cheap, very alcoholic drinks **[See posters for details]**. Consequently, this policy of minimum pricing is believed to have the greatest effect on people who typically drink cheap alcohol the most (i.e. **young binge drinkers**, and **heavy low-income drinkers**), and who also normally suffer most from alcohol-related problems (e.g. liver cirrhosis, violent behaviour).

**Minimum pricing is also hoped to encourage drinkers to switch to weaker wines and beers.** This is because drinks containing a greater number of alcohol units will have a higher minimum price than those with less (e.g. super-strength lagers will cost more than weaker lagers – **SEE LAGER POSTERS**).

In addition to this, **the introduction of a minimum price per unit of alcohol would also put an end to the sale of heavily discounted alcohol in supermarkets & off-licences** (e.g. “BUY ONE GET ONE FREE”). As a result of this, off-licence and supermarket prices would not differ significantly from to those in pubs and nightclubs, which at present they do.

Research has shown that an increase in the price of alcohol leads to reductions in alcohol consumption, binge drinking, alcohol dependence, and the problems associated with these. It is estimated that a minimum price of 40p per unit would save over 1,100 lives per year, and 3,000 lives with a minimum price of 50p per unit.

What are you your **immediate thoughts** about this idea of a minimum price per unit of alcohol?

What do think about the idea of minimum alcohol pricing?

Do **like** the idea of a minimum price per unit of alcohol?

Do you think it will **work**?

Do you think that introducing minimum pricing will actually reduce how much people drink?

What do you think are the **possible outcomes** of a minimum price policy?

- SIX MONTHS?
- A YEAR?
- 5 YEARS FROM NOW?

How do you think a minimum price per unit might influence **your drinking**?

What concerns might you have about minimum alcohol pricing?

Do you think this is a **fair way to deal with alcohol problems**?

**Who** do you think will be most influenced by price increases?

Do you think minimum pricing will affect poor and rich people differently?

What impact do you think minimum alcohol pricing will have on….

- Underage drinking?
- Heavy drinkers?
- Moderate drinkers?

What effect, if any, do you think minimum price policy might have on the **problems associated with people drinking too much** (e.g. crime, social disorder, hospital admissions, etc)?

In general, what do you think about government intervening with peoples’ drinking behaviour?

Is alcohol different to other commodities? Would you continue to drink excessively regardless of any price increase?

Are you favour of minimum alcohol pricing or not? **If not, why not?**

**How could the introduction of a minimum price policy be made more acceptable to you?**

**If you were in government, what would you do to deal with the problems associated with alcohol?**

## Additional file 3: Appendix 3 – Additional quotations

(To be Made Available as Supplemental Material Only)

Additional quotations derived from the qualitative inductive thematic content analysis of the focus group data are presented below. The quotations are organized under the primary and secondary thematic headings.

1. Will a minimum price per unit be effective in reducing alcohol consumption?

1.1 - A minimum price is unlikely to be effective at all

1.1.1 “Where there’s a will, there’s a way”

*I don’t think it’s going to work… Necessity is the mother of invention, so if people want alcohol, they’ll find a way to get it... there’s plenty of ways to get money to get alcohol.*

[FG3 – Sixth-form student]

*I think you’d just find another way of getting cheap alcohol. Something will be brought in... there will always be a way to get cheap stuff I should think.*

[FG6 – University student]

*I think if the prices went up, I’d find a way to still buy it cheap and carry on.*

[FG7 – University student]

*One way or another, it won’t stop people drinking if they want to.*

[FG11 – Blue-collar worker]

*It wouldn’t work… people will always find a way round it.*

[FG17 – Unemployed]

*If they want to drink, they’ll find a way to do it. So personally I don’t think it’ll make much difference at all.*

[FG22 – Older adult]

*If you want something you’re going to get it aren’t you? Regardless of what the price is, if you want that you’re going to get it.*

[FG23 – African-Caribbean]

*I think that people will just cut back on other things if they can’t afford to drink.*

[FG2 – Sixth-form student]

*People will still keep on drinking, they might cut down on their other expenses.*

[FG4 – University student]

*I think people will just find ways round it, with booze cruises, home brews, and bootlegging.*

[FG13 – Office worker]

*If they put the price up, then people [will] start to home brew... They will start making it at home.*

[FG24 – South Asian]

*But they’ll save on something else, in order to compensate for alcohol… If people want to drink, they’ll cut down on how much they spend on their shopping bill, how much they gamble, or whatever... how much they drive their car, they’ll cut down.*

[FG25 – South Asian]

1.1.2 - Minimum pricing won’t work for heavy & dependent drinkers

*I don’t think it’s going to affect the people that actually drink loads…. My auntie is an alcoholic and I don’t think anything will stop her buying her alcohol. So I don’t think it would make a difference if they raise the prices…So actually [for] people who need help, I don’t think it’s going to make a difference.*

[FG1 – Sixth-form student]

*The people who are taking it to stupid extreme and ending up in hospital, I would be tempted to call them addicts… when you’re drinking that much, that frequently, and it’s affecting you like that, and addicts will get what they’re [going] for regardless of the price.*

[FG6 – University student]

*I don’t think it’ll work on the people who have a drink problem because they will find a way to do it.*

[FG11 – Blue-collar worker]

*I think the people who are at the extreme end of the problem, who are the heavy drinkers, who perhaps even drink so much that they’re going to get ill or that they suffer from alcoholism, it doesn’t matter how much it costs, they’ll just find a different way of getting the money to pay for it.*

[FG13 – Office worker]

1.1.3 Perceived failure of previous price control policies

*Smoking is a social thing like alcohol… So if you were judging it on what’s happened with cigarettes, then this isn’t really going to work very well.*

[FG3 – Sixth-form student]

*I mean the comparison is, for me, cigarettes. I mean cigarettes are ridiculously expensive, but people still pay the money because they want cigarettes… but if they want it enough then they’ll just keep [drinking], they’ll just be forced to pay the extra money.*

[FG8 – University student]

*I doubt that it would work for the same reason that smoking continues, and I think possibly even drinking’s more accepted in a way than smoking. So it stands less of a chance.*

[FG12 – Office worker]

*I think they will [keep buying alcohol] whatever price it is, they will buy it. It’s like when cigarettes kept going up and up, they still kept buying them... it didn’t stop them.*

[FG20 – Older adult]

1.2 A minimum price will only have a limited effect

*It might have a small impact but probably not worth all the effort that they’ll go through to put it through.*

[FG7 – University student]

*It could work, have a slight impact, but I don’t think anything like what they would want...It wouldn’t solve the problem.*

[FG12 – Office worker]

*What difference would it make if the majority of people reduce their alcohol consumption by one or two units a week, or even three or four units a week?*

[FG13 – Office worker]

1.2.1 A minimum price will only change people’s choice of drinks

*I think that people would just be clever with what they bought, and try and maximize by just swapping drinks.*

[FG7 – University student]

*It will affect drink choices, you’d just switch from say beer to wine, and in the long run it won’t have that much effect I think. So it’s not really a good idea to raise the prices.*

[FG7 – University student]

*Instead of buying the cheap strong beers, they’ll move on to something like a bottle of scotch, or something which is, hasn’t changed by that much [in price]… So I think it’ll just transfer the problem from people drinking cheap cider, from drinking cheap scotch, and then they’ll be having the same kind of debates in ten years about putting the price of scotch up and it’ll kind of go on from there.*

[FG8 – University student]

*If you just make it on the high alcohol, like Diamond White or whatever, instead of buying that they’ll just buy a bottle of cheap whiskey, they’ll buy a couple bottles of that, instead of one bottle of that, which would give them the same effect, there’s no difference.*

[FG11 – Blue-collar worker]

*I don’t think it would solve the problem because these people [binge drinkers]…. would then go to the cheaper one and they buy more because it’s cheaper.*

[FG19 – Older adult]

*I just think that they [drinkers] will probably just switch to something else.*

[FG25 – South Asian]

1.2.2 A minimum price will only reduce binge drinking frequency

*[People will] probably drink less often, but more heavily when they do drink.*

[FG2 – Sixth-form student]

*I’d just save the money I would have been spending on casual drinking, and just save up for the big nights.*

[FG5 – University student]

*People are drinking slightly more but on fewer occasions when they do, [if they] drink slightly more... the alcohol problems, you know, hospital admissions and stuff could shoot up, you never know.*

[FG9 – University student]

*I think it might make binge drinkers not binge as many times in a month maybe…if they did every single weekend, maybe they’ll only do it twice a month.*

[FG11 – Blue-collar worker]

*Maybe people will drink the same amount less often… people would save-up their units for one night maybe, rather than two.*

[FG13 – Office worker]

*You’re more likely to binge because they can’t do it as often as what they used to... more likely to binge and binge worse than what they what they used to.*

[FG15 – Unemployed]

1.2.3 A minimum price will only affect young people

*I think it might only work for young people though…teenagers prefer to drink the cheaper stuff, so it’ll only affect them really.*

[FG3 – Sixth-form student]

*It might [work], but I’m not sure, for just some groups of people, for example, very young people… [but] not for everybody.*

[FG15 – Unemployed]

*I think it’s only going to affect people who are under eighteen, who are trying to drink as much as they can but for as cheap as possible.*

[FG27 – Rural community]

*It could reduce underage drinking a bit, but not anybody else.*

[FG28 – Hazardous drinker]

1.2.4 A minimum price will only lead to short term reductions

*My personal use would probably go down, and then probably go back up again [after I] got used to the price change.*

[FG1 – Sixth-form student]

*I think people six months down the line people would just be used to the new prices, everyone just accepted the new prices... People have sort of adjusted how they spend their money, people [will] probably still drink the same… and the government [will] probably start trying to look for a new way to try and address the binge drinking issue.*

[FG7 – University student]

*To be honest, I don’t think it’s going to affect anyone in the long term. Like, initially there’ll be outcry, and people will be like, oh I can’t afford White Lightning, and then there’ll just be a shift, and they’ll be exactly the same. I don’t think it’s going to affect anyone long term at all.*

[FG13 – Office worker]

I don’t think it’s going to make a difference. I think people will just get used to that price and carry on.

[FG26 – South Asian]

1.2.5 A minimum price will only have long term effects

*It won’t really affect our generation, it’ll affect the next generation...you won’t see the effects in this generation of binge drinking, you’ll see the next generation not really drinking that much.*

[FG2 – Sixth-form student]

*I think it’ll work. I think the impact on the generations now, would be minimal to average. I think going forwards, [for the] next generation and the ones after that, I think it’ll just become accepted and that’s how it is, and I think that’ll be the biggest benefit.*

[FG11 – Blue-collar worker]

*I actually think this is probably something that would work if you change the price, but not in our generations. I think may be two generations down the line... But for our generation of people in here, I don’t think it’ll have much of an effect.*

[FG11 – Blue-collar worker]

*I don’t think it’ll make an amazing difference… It will have an effect, but an eventual effect, not an immediate effect.*

[FG26 – South Asian]

2. Do people like the minimum price per unit of alcohol proposal?

2.1 - Dislike for a minimum price per unit of alcohol

2.1.1 Punishing the moderate or ‘sensible’ drinker

*[It] seems to be punishing people… people [that] haven’t done anything wrong, there’s some people who are going out and getting absolutely drunk, and ending up in hospital, and that is a strain on our society. But a lot of people are just drinking socially and having a good time, we’re not posing a problem to our country at all. So I don’t see why we should be punished by high prices.*

[FG6 – University student]

*It doesn’t directly target the problem, it targets the country as a whole. The country as a whole isn’t a problem, it’s a proportion of the population who are the problem.*

[FG7 – University student]

*I’m a responsible drinker, why should I get penalised for people that can’t control themselves?*

[FG11 – Blue-collar worker]

*It is penalising the majority for the minority’s problems.*

[FG12 – Office worker]

*Personally I feel a bit offended that it would be people like myself that’ll be having to pay some more money… I just feel the principal of it is unjust, when it’s sensible drinkers who are not causing the problem, but they’re still going to end up having to pay for it.*

[FG13 – Office worker]

2.1.2 - A restriction of the personal freedom to drink alcohol

*I think its restricting people’s choice.*

[FG4 – University student]

*I don’t agree with it. I don’t see [why] the government should tell us what we can do, and what we can’t do.*

[FG11 – Blue-collar worker]

*I think we live in too much of a nanny culture as it is... there are too many rules and regulations about what to do, what not to do. If people want to slowly kill themselves because of alcohol, [they can]. Yes, it has a huge burden on the NHS and therefore society as taxes as a whole, but at the end of the day this isn’t necessarily going to stop them… I think people should be left alone.*

[FG12 – Office worker]

*I just don’t like the idea that the government trying to control your life, that’s something I don’t like.*

[FG25 – South Asian]

2.1.3 Minimum pricing unfairly targets the poor

*I think they’re intervening in the wrong way... well, it’s bad for the poorer people, because it’s not going to affect people that are really rich really at all, is it? So I think they’ve got this the wrong way round.*

[FG3 – Sixth-form student]

*But there are people on low incomes, if they want to go and have a drink that’s going to cost them a lot of money. They’re going to be less well off, and they probably will still drink to the same extent, and will have less money, maybe for their children or whatever.*

[FG9 – University student]

*The only sort of downside for me is that people on low-incomes, they should be able to a drink, and they shouldn’t be excessively penalised because of this.*

[FG12 – Office worker]

*It targets low-income people basically... So in that sense, no, it’s not fair.*

[FG14 – Office worker]

2.1.4 - “There’s more to alcohol reduction than price”

*I’m not really in favour of it because it doesn’t address the core issues that make people drink in the first place.*

[FG3 – Sixth-form student]

*I think it just seems quite a simplistic way to sort of address the issue… it’s part of the culture, drinking… And to just say, oh people drink because it’s cheap, it doesn’t really address the sort of deeper, cultural issues.*

[FG7 – University student]

*It’s almost a cultural thing, and we’re trying to put a sticky plaster on it... by doing the price thing, and it’s a deeper thing than that. And it is going to penalise the people who like a drink, and it’s not a problem for them.*

[FG12 – Office worker]

*It’s not addressing the proper issue is it? Why do people drink too much? It’s not answering that… It’s not looking at society as a whole, and the problems that are causing it.*

[FG13 – Office worker]

2.1.5 - “There must be a better way than this”

### There are other ways to deal with it if they really, really wanted to deal with it.

[FG13 – Office worker]

*I personally don’t mind that government thinking about helping people to reduce our drinking… But I think that they should think of better ways.*

[FG15 – Unemployed]

### Well the government seem to think that that is the answer to everything… putting prices of things up. They don’t look at other ways round it.

[FG19 – Older adult]

### I’m not in favour, because I think there’s other ways of dealing with the problem.

[FG20 – Older adult]

*There’s got to be a better way than this.*

[FG20 – Older adult]

2.1.6 - Suspicion of government motives

*I’m not in favour, because it’s a sneaky way of getting more money out of people for the treasury.*

[FG13 – Office worker]

*[I am] not in favour of it. I think they’re not fixing the underlying problem, they’re just putting a plaster on it. I think it’s probably more of a publicity stunt and a way to make money than actually solve the problem.*

[FG13 – Office worker]

*It’s a tax in everything but name.*

[FG22 – Older adult]

*It’s just another one of the things that they want us, people to know about, to make us think that they’re doing something.*

[FG23 – African-Caribbean]

2.2 Support for the introduction of a minimum price per unit

2.2.1 The need for action to curb excessive alcohol consumption

*I think it’s quite a big problem, and something needs to be done to sort it out.*

[FG2 – Sixth-form student]

*At least they’re trying to address the problem somehow.*

[FG7 – University student]

*But if they go ahead with it, I don’t think I’d complain because I know they’ve got to do something.*

[FG11 – Blue-collar worker]

*So it’s a start, and I certainly would be for anything that’s a positive and working towards that [reducing alcohol consumption].*

[FG14 – Office worker]

*I’m in favour simply because something’s got to be done.*

[FG22 – Older adult]

2.2.2 The prospect of improved public health

*I think it’s a good idea... it’s going to stop younger people drinking as much.*

[FG1 – Sixth-form student]

*I’m in favour of it... For the simple reason you’ve got to think of the younger generation. I’m not bothered about the alcoholics, they’ve been drinking for years and years. It’s the younger generation that I think we’ve got to educate... and if by putting it up a few pence stops even one of them buying it and drinking it’s worth it.*

[FG11 – Blue-collar worker]

*Yes. I am in favour of it, because hopefully it will reduce the people that are drinking a lot. Hopefully it’d give them a better life, so [they’re] not so reliant on the NHS.*

[FG18 – Older adult]

*I am in favour of it… stopping underage drinking, and obviously getting the people that are alcoholics to reduce their consumption.*

[FG28 – Hazardous drinker]

2.2.3 “It doesn’t bother me, I don’t drink that much”

*I feel quite indifferent to be honest... somehow I don’t feel like it’ll be affecting me personally with my [drinking]. I’m already paying the increased price at the moment, so... I wouldn’t support it, but I wouldn’t oppose it either.*

[FG8 – University student]

*I’m in favour of it because I’m not a drinker.*

[FG16 – Unemployed]

*I’m not too bothered to be honest, because I don’t really drink much and I don’t really care what everybody else does.*

[FG25 – South Asian]

3. A minimum price policy might make matters worse

*I’m not in favour. I think it’ll make things worse.*

[FG15 – Unemployed]

*If the prices go up, I think it’s going to make it worse.*

[FG15 – Unemployed]

*I just think it’s going to cause more problems than it’s going to solve...I think it’ll make it worse.*

[FG16 – Unemployed]

3.1 Increases in crime

*There will not be any long-term benefit... because it’ll cause problems elsewhere. People [who] need a drink, they’ll get it by other sources. If they can’t afford it they’ll go out and steal, whether it’s the wines and spirits themselves, or steal money, or rob, they’ll do whatever.*

[FG10 – Blue-collar worker]

*I think there might be a lot more crime because I think people might steal what they can’t afford.*

[FG18 – Older adult]

*If you increase the price for those who are dependent upon alcohol for their fun, for their buzz or whatever, will fund it somehow. So therefore crime will go up, whether it’s petty crime or major crime, they will fund it somehow.*

[FG22 – Older adult]

*Crime will go up, definitely… because I think people who are alcoholics, they need to drink…if you put the price up, they need the money, the crime will go up.*

[FG24 – South Asian]

3.2 Increases in drug abuse

*It’s working out now that a line of cocaine is cheaper than a pint of beer from a pub... So [people] might go on to some other form of drugs instead because it would be cheaper.*

[FG1 – Sixth-form student]

*There might be more drug usage… because people will find alternatives, an alternative to alcohol... Alcohol gives them a feeling of happiness and stuff, so they’ll look for something else that gives them that feeling.*

[FG2 – Sixth-form student]

*If you’ve got a mind-set where you actually want to go and get off your head, the cheapest way to do that might be to go and get some cocaine or something.*

[FG13 – Office worker]

*Well they’ll start taking cocaine, because cocaine will be cheaper than buying alcohol. So then you’ve got a cocaine problem.*

[FG19 – Older adult]

*They will follow the cheapest commodity, whether it be drugs [or alcohol] to get the same buzz.*

[FG22 – Older adult]

3.3 Negative economic impact

*I think that it’ll cause a lot of problems with alcoholics on low income, because they’ve got an illness, they can’t help themselves, they need that alcohol so they’re just going to end up in more debt.*

[FG2 – Sixth-form student]

*[It] would probably reduce revenues for the bars and stuff, which would be a kind of big disadvantage against this.*

[FG4 – University student]

*It would probably end up putting people out of business.*

[FG12 – Office worker]

*I think if anything, it’ll just cause financial problems because a lot of the people who drink heavily, for example, people who have no houses, students, and like people who are unemployed maybe, are stressed so they drink a lot. They haven’t got any money anyway, and they’re still doing it. So students will just get more overdrafts, get more loans and get in more debt, as will everyone else who’s got no money and is still [drinking]... It’ll just cause more financial difficulties.*

[FG13 – Office worker]

4. Maximizing the acceptability of a minimum price policy

4.1 Minimum price as part of a broader policy ‘package’

*Yeah, [I’m in favour] if this is combined with other schemes then it will work very well. But if this is the only thing, they don’t do anything else, like they don’t help in education, like, teaching young children about alcohol, like, even the units thing at a young age… then the programme will work, but otherwise on its own it won’t, I don’t think it’ll do that.*

[FG2 – Sixth-form student]

*I think potentially it could [work], yeah. But I don’t think just this measure would make a difference... I think there needs to be a combined approach, like the pictures that you get on cigarette packets now, you know, obscene throat cancer and stuff like that. I think it needs to be a joined-up approach.*

[FG11 – Blue-collar worker]

*I think I would be in favour of it, but not on its own. I think it’s one tool in the armoury so to speak.*

[FG12 – Office worker]

*It’s not just a one thing, we don’t feel it’s all about the price, so just doing this to the price wouldn’t necessarily change it... you’d want it to then also provide other activities, or to help other people that are suffering from this, of the NHS or other things to do… You’d want it to come as a package, not as [if]this is going to solve everything.*

[FG14 – Office worker]

*I think it could possibly work but it would have to work in conjunction with other things. It couldn’t do it on its own, they’d need to have education with it.*

[FG17 – Unemployed]

4.2 Funding for other interventions

*I’m against it. I don’t think it’ll work, and I’d probably be more for it if I thought the profits were going to go to the NHS, and the police.*

[FG1 – Sixth-form student]

*I don’t see that’s going to help the situation. I’m also a bit suspicious as to where the money would be going... If it was going in to helping people with alcohol-related issues then fair enough. If it was going into education in school, maybe setting-up community centres and youth facilities so that kids weren’t bored and hanging round the streets, and didn’t feel the need to go out and have two bottles of cider between four of them on a nightly basis, then it’s a good idea.*

[FG12 – Office worker]

*I think that this proposal alone wouldn’t work, therefore I’m not in favour of it. But I think there’s a possibility that they could use the extra money they’re making on the alcohol to put directly into other ways of tackling the same issues, and then the two combined could work, but this proposal alone I wouldn’t agree with.*

[FG13 – Office worker]

*But if they did [that] with the extra money that they’re making from per unit, if they ring-fence it and then educate and rehabilitate, then it’d work. Then it’d be worth it.*

[FG17 – Unemployed]

*It’s got to be packaged right, if he says this is where the money’s going to be going, you’ll see more policemen, for instance, on the beat, and we’re going to clamp down on the on-street drinking, then they might take the public with him.*

[FG18 – Older adult]
